# Preliminary development of a questionnaire measuring patient views of participation in clinical trials

**DOI:** 10.1186/s13104-019-4724-z

**Published:** 2019-10-21

**Authors:** Judith Arnetz, Sukhesh Sudan, Courtney Goetz, Bengt Arnetz, Laura Gowland, Suzanne Manji, Samiran Ghosh

**Affiliations:** 1 Department of Family Medicine, Michigan State University College of Human Medicine, 15 Michigan Street NE, Grand Rapids, MI 49503 USA; 2 Department of Emergency Medicine, Wayne State University School of Medicine, Detroit, MI USA; 3 Department of Psychiatry, Wayne State University School of Medicine, Detroit, MI USA; 4 Department of Family Medicine and Public Health Sciences, Wayne State University School of Medicine, Detroit, MI USA

**Keywords:** Questionnaire, Patients, Clinical trials, Psychometric evaluation, Factor analysis

## Abstract

**Objective:**

This study aimed to develop a questionnaire for measuring patient perceptions of participating in clinical trials. Development was based on earlier research on patient views of involvement in medical care and a literature review. Patients were recruited from an ongoing clinical trial focused on cardiovascular illness and from an outpatient psychiatry department. Factor analysis was conducted on a pilot version of the questionnaire in 2016 and on a revised version in 2017.

**Results:**

A total of 53 patients were recruited for the pilot study and 55 were recruited for the main study, substantially below the goal of 100 participants. Factor analysis revealed six factors measuring aspects of patients’ perceptions of participating in clinical trials, including motivation, risks and benefits, the nature of the trial itself, and practical considerations, such as cost and convenience. Inter-scale correlations ranged between 0.06 and 0.64, indicating acceptable scale independence. Reliability scores (Cronbach’s alphas) ranged from 0.62 to 0.85. Factor analysis results were somewhat unstable, with shared variance for several items across scales. This is likely due to the small sample sizes. In larger, more diverse patient samples, this questionnaire can be useful for measuring and incorporating patients’ views into the design and execution of clinical trials.

## Introduction

Randomized controlled clinical trials continue to be considered the gold standard for establishing efficacy of medical treatment. Yet recruitment of patients for participation in trials is a continuous challenge, causing delays in both research studies [1] and scientific discovery [2]. Factors that have been associated with patient participation include communication with providers and family members [2, 3], preference for alternative treatments, convenience [4], medical history, and insurance [4, 5]. A meta-analysis and systematic review found that barriers to participation in clinical trials were related to the study protocol, patient factors, or physician factors [6]. These studies concerned oncology patients, and it is not known whether the same factors apply for patients with other medical conditions. In general, a better understanding of patients’ views of participating in clinical trials would provide a critical missing piece in the design, conduct and successful execution of clinical trials.

The current study was part of a larger project examining the use of Bayesian statistical methods in non-inferiority trials [7]. The aim was to develop a questionnaire for measuring patient perceptions and expectations of participating in clinical trials, with the intention to understand how these perceptions could be converted into probability distributions and integrated into the design of clinical trials. This brief report presents preliminary factor analysis results from the development of the questionnaire.

## Main text

### Methods

#### Questionnaire development

Development of the questionnaire was based on earlier research on patient perceptions of involvement in medical care [8] and a literature review. Much of the existing research on patient views of participating in clinical trials has focused on cancer patients [3, 6, 9–13], while a few studies examined cardiology [14] and heart attack patients [15] or the general public [9, 14]. While most studies utilized questionnaires [3, 9, 11–13, 15–17], only three reported psychometric properties of their instruments [9, 11, 17]. All of the referenced studies were at least 10 years old.

We developed a 44-item pilot questionnaire. Thirteen items covered demographic/personal information. Six items adapted from a previously-validated questionnaire on cardiology patients’ views of involvement in hospital care [8] measured patients’ views of what involvement in clinical trials meant to them. The remaining 24 items were derived from the literature review. Two studies on patient motivation to participate in clinical trials [3, 13] included questions based on questionnaire items originally published by Daugherty et al. [3] that the research team deemed potentially relevant to our study. The 24 items, identified by two members of the research team, were reviewed for content validity by a team of researchers and healthcare professionals as to how well they represented the constructs they were intended to measure. The questionnaire items were derived from studies using focus groups [8] and interviews [3] with patients, which supported their relevance to the patient experience.

All 24 items were scored on a four-point scale from either disagree completely [1] to agree completely [4], or not at all important [1] to very important [4]. One open-ended item enabled patients to share any additional thoughts about participation in clinical trials and 4 additional questions asked for perceptions about the survey itself (e.g., length, clarity of questions). Based on psychometric analysis of the 2016 pilot questionnaire, described below, a final, 41-item version of the questionnaire was developed and administered in 2017 (Additional file 1).

#### Setting and study participants

The questionnaire was developed for use with any patient group, regardless of diagnosis. The goal for this study was therefore to recruit two different groups of patients. Based on feasibility and availability, we focused on those seeking care for either cardiovascular or mental health conditions. Participants with heart failure were recruited in Detroit, Michigan from an ongoing clinical trial, the Acute Heart Failure Registry of Emergency Department Patients study, part of the Emergency Medicine Research and Outcomes Consortium (EMROC). Patients with mental health conditions were recruited from an outpatient university psychiatry department in Detroit.

#### Data collection

Patients for the pilot study were recruited in an urban hospital emergency department (ED), as well as in the Department of Psychiatry of a participating university. ED patients were recruited in conjunction with their agreement to participate in the EMROC study, while mental health patients were recruited when coming to their scheduled appointments at an outpatient university psychiatry department. Patients for the main questionnaire study were recruited only in the hospital emergency department. Data collection for the pilot study took place between January and February 2016, with a total of 53 patients agreeing to respond. Of these, 24 (45%) were from psychiatry and 29 (55%) were cardiovascular patients. Data collection for the main questionnaire study took place between April and July 2017, with 55 patients recruited. All respondents willing to provide their mailing address received a $15 gift card for the pilot study, and a $25 gift card for the main study. Respondents were assured that their personal information would be kept separate from their questionnaire responses.

#### Data analysis

Analysis of both the pilot and the main questionnaires utilized exploratory factor analysis to determine whether any scales could be created. Procedures were identical for both questionnaires. In a first step, a correlation matrix was run to study the inter-item correlations of all non-demographic variables. The matrix was comprised of 30 items for the pilot questionnaire and 27 items for the main questionnaire. Bartlett’s test of sphericity and the Kaiser–Meyer–Olkin measure of sampling adequacy (KMO) were used to assess the factorability of the correlation matrix. To justify factor analysis, Bartlett’s test should be significant and KMO values should exceed 0.60 [18]. Next, exploratory factor analysis was used to examine the structure of the relationships between questionnaire items and to determine whether subscales (factors) could be created. Principal component analysis using Varimax rotation and scree plots was used to extract the factors. Correlation analysis was used to examine the independence of the factors as a measure of construct validity, and Cronbach’s alpha was used to measure each factor’s internal reliability. Second-order factor analysis was then used to see whether forcing the factors to a limited number of dimensions improved the overall explained variance. Three criteria were used in creating subscales: [1] scales should have patient relevance based on content validity; [2] item loadings should be ≥ 0.40 [19]; and [3] reliability coefficients should be ≥ 0.70. Due to the small sample sizes, no tests of either questionnaire’s convergent or discriminant validity were conducted.

### Results

#### Pilot questionnaire

Patient respondents (n = 53) were primarily African-American (68%), White (30%), and female (55%), with a mean age of 51.09. The initial exploratory factor analysis resulted in the extraction of 8 factors with Eigen values ≥ 1 that together explained 74.75 of the overall variance. The KMO measure of sampling adequacy was 0.475, which is considered inadequate [18]. However, Bartlett’s test of sphericity was significant (χ^2^ = 1222, p = 0.000), which justified proceeding with the analysis. All coefficients that loaded below 0.40 were suppressed. Since several of the 8 factors were only comprised of 2 items and there was shared variance between factors, attempts were made to force the items to a 7- and a 6-factor solution. The 6-factor solution was best, explaining 65% of the total variance, with an overall alpha of 0.82 (Table 1). Three items did not factor into any of the subscales and were therefore excluded, reducing the total number of items to 27. The 3 excluded items were the following statements that patients were to agree/disagree with or rate the importance of: “The patient has the main responsibility for his/her future health” (item 19); “Who are in the research team” (item 29); and “That you could withdraw from the clinical trial at any point in time at your will without affecting your future care” (item 41).

**Table 1:** Loadings of variables on factors (italic print) emerging from rotated component matrix

Item no.	Abbreviated item label	Factors						Communalities
		1	2	3	4	5	6	
14	Receive clear information			*0.66*				0.46
15	Can ask questions			*0.91*				0.88
16	Express views	*0.82*						0.76
17	Involved in discussions	*0.94*						0.90
18	Involved in decisions	*0.63*						0.51
19	Responsibility for health			0.43				0.37
20	Type of treatment	*0.64*						0.46
21	Purpose of treatment	*0.54*						0.54
22	Side effects		*0.84*					0.74
23	Risks		*0.73*	0.52				0.91
24	Benefits		0.60		*0.64*			0.78
25	Cost-free treatment	*0.77*						0.63
26	Pain/discomfort	0.64	*0.68*					0.88
27	Quality of life				*0.83*			0.70
28	Part of medical research	*0.58*						0.64
29	Research team members			0.47		0.42		0.58
30	Understanding trial aim	*0.65*						0.68
31	Understanding—help you now			*0.84*				0.74
32	Understanding—help others now	0.70					*0.45*	0.80
33	Understanding—help you in future						*0.78*	0.65
34	Understanding—help others in future						*0.88*	0.80
35	Compensation					*0.75*		0.66
36	Out of pocket costs		*0.74*			0.52		0.84
37	Convenient					*0.68*		0.57
38	Travel		0.57			*0.59*		0.72
39	Need help in participation					*0.55*		0.52
40	Confidential				*0.80*			0.66
41	Withdraw							0.22
42	Ethically approved	*0.64*						0.48
43	Person to contact during trial		*0.75*					0.60

Communalities are estimates of the variance accounted for by each variable in the factor solution

Correlations between scales were examined using Spearman’s rho (Table 2). Inter-scale correlations ranged between 0.06 and 0.64, indicating generally acceptable scale independence. The highest correlation between risks and cost and convenience (0.64, p < 0.001) indicates some overlap between those scales.

**Table 2:** Bivariate correlations (Spearman’s rho) between the patient questionnaire subscales (n = 53)

	1	2	3	4	5	6
1. Motivation	–					
2. Risks	0.37**	–				
3. Understanding purpose	0.21	− 0.13	–			
4. Benefits	0.31*	0.32*	0.23	–		
5. Cost/convenience	0.23	0.64***	0.06	0.24	–	
6. Contribution to health	0.33*	0.11	0.34*	0.11	0.10	–

* p < 0.05, ** p < 0.01, *** p < 0.001

#### Main questionnaire

Patient respondents (n = 55) were primarily African-American (95%), 51% were male, and the mean age was 58.85. Exploratory factor analysis included all 27 items and resulted in a 5-factor solution explaining 77.8% of the variance. KMO was 0.749 and Bartlett’s test was significant (χ^2^ = 1780, p = 0.000). Multiple iterations were conducted, including forcing the items to 4, 6, and 7 factors. Each solution had substantial shared variance between items. We ran reliability estimates (Cronbach’s alpha) on the 6 scales that had been identified in the pilot study, even though they were not supported by the factor analysis in this second phase. Reliability estimates were 0.78 or higher for the main questionnaire and ranged from 0.62 to 0.85 for the pilot data (Table 3).

**Table 3:** Patient questionnaire subscales with mean scores and reliability estimates (Cronbach’s alpha)

Subscale	No. of items	Score range (min–max)	Pilot study		Final study	
			Mean (SD)	Cronbach’s alpha	Mean (SD)	Cronbach’s alpha
Motivation	9	(9–36)	34.70 (2.52)	0.85	34.20 (3.88)	0.92
Risks	5	(5–20)	19.09 (2.10)	0.85	18.73 (2.49)	0.83
Understanding trial purpose	3	(3–12)	11.81 (0.62)	0.67	11.49 (1.45)	0.83
Benefits	3	(3–12)	11.68 (0.78)	0.78	11.40 (1.53)	0.85
Cost and convenience	4	(4–16)	13.98 (2.07)	0.62	13.58 (2.87)	0.78
Contribution to health	3	(3–12)	11.38 (1.04)	0.67	11.60 (1.38)	0.96
Total number of items	27					

### Discussion

The aim of this study was to develop a questionnaire for measuring patient perceptions and expectations of participating in clinical trials. Patients with acute heart failure were recruited once they had agreed to enroll in an ongoing clinical trial, a method that has been used in previous cancer therapy trials [3, 13]. Preliminary analyses identified six scales measuring various aspects of patients’ perceptions of participating in clinical trials, encompassing factors concerning motivation, risks and benefits of participation, the nature of the trial itself, and practical considerations, such as cost and convenience. Our findings that patients are motivated to participate by expectations of potential health benefits are in line with previous studies of cancer patients [3, 13]. However, contributing to other patients’ health, also a factor in the current study, was less important to the cancer patients [3, 13], although altruism was identified as a factor in earlier research of myocardial infarction (MI) patients, as was convenience [15].

## Limitations

Building on the pilot analysis, our goal was to replicate the factor analysis in a larger sample. However, due to time and resource constraints, the research team was only able to recruit 55 patients from the EMROC study for the second phase of questionnaire analysis. Based on the pilot study data, a sample size of 147 would be required to detect significant differences in the Motivation subscale between male and female patients with a power of 0.80 and a type I error rate of 0.05. With only 55 participants, our main study was therefore clearly underpowered.

Preliminary analyses indicated good internal consistency of the scales, measured with Cronbach’s alpha, but factor analysis results were somewhat unstable, with shared variance for several items across scales. This is likely due to the small sample sizes. Despite much discussion in the scientific literature regarding minimum sample sizes for factor analysis, it is suggested that a greater number of variables per factor and higher levels of communality usually allow for smaller sample sizes [20]. Nevertheless, results of these analyses suggest a need to re-evaluate with additional, larger samples of patients. Larger samples would also enable more robust validity testing, the aim of which would be to establish that the scales generated by the factor analysis measure what they intend to measure. Discriminant validity would be examined by studying the associations between subscales, patient demographic characteristics, and patient diagnosis (psychiatric vs. cardiovascular) in order to test each scale’s ability to capture differences between groups. Finally, data were collected from a large, urban hospital and the demographics of patient respondents may not be representative of the general population. This questionnaire therefore needs to be tested in both larger and more diverse populations. Despite these study limitations, this questionnaire can be useful for measuring and incorporating patients’ views into the design and execution of clinical trials.

Designing clinical trials to be more patient-centered could potentially increase the likelihood of patient recruitment and even of trial effectiveness. However, quantifying the patient perspective and utilizing it to improve trials is in its infancy. These preliminary results reveal several domains of importance to patients when considering participation in clinical trials. While further psychometric testing is needed, this questionnaire represents an important first step in incorporating patient views into the design and recruitment process of clinical trials.

## Supplementary information

**Additional file 1.** Patient_questionnaire.

## Data Availability

The data sets used and/or analyzed during the current study are available from the corresponding author on reasonable request.

## Abbreviations

EMROC: Emergency Medicine Research and Outcomes Consortium
ED: Emergency Department
KMO: Kaiser–Meyer–Olkin measure of sampling adequacy
PCORI: Patient Centered Outcomes Research Institute
