# Supplementary figures and images for: Preliminary development of a questionnaire measuring patient views of participation in clinical trials
Source: BMC Res Notes. 2019 Oct 21;12:667. doi: 10.1186/s13104-019-4724-z (PMC6805544; doi:10.1186/s13104-019-4724-z)

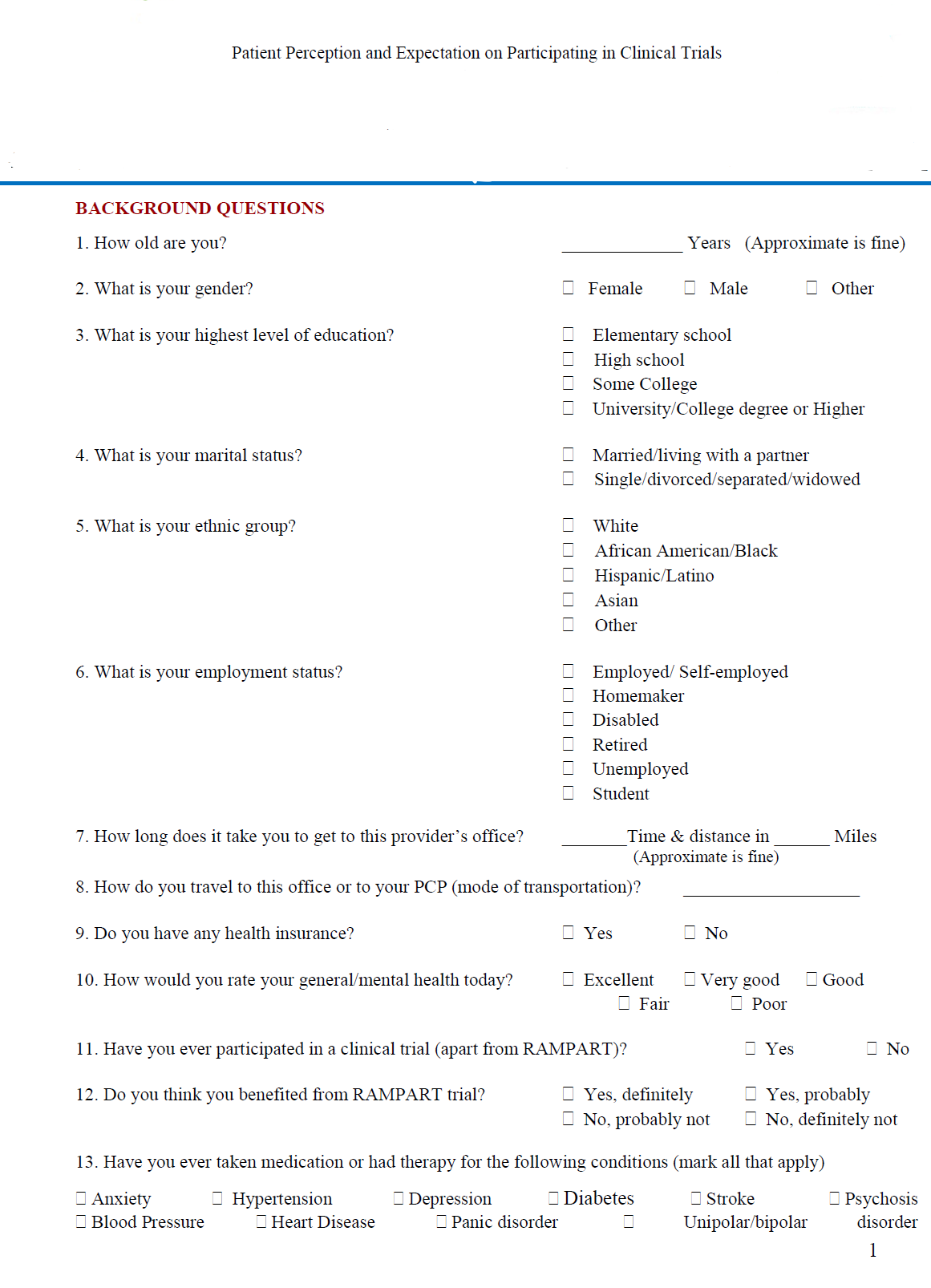

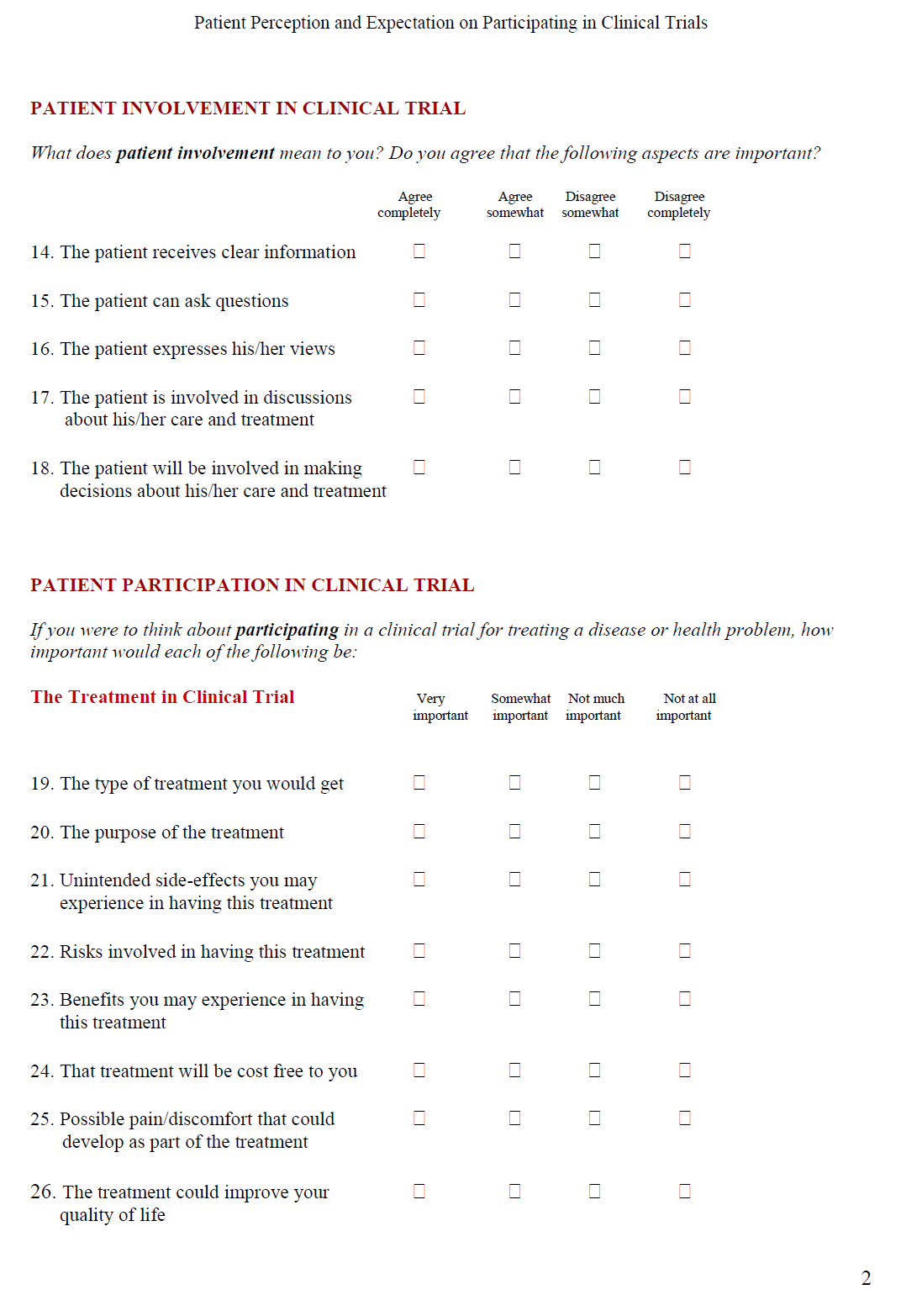


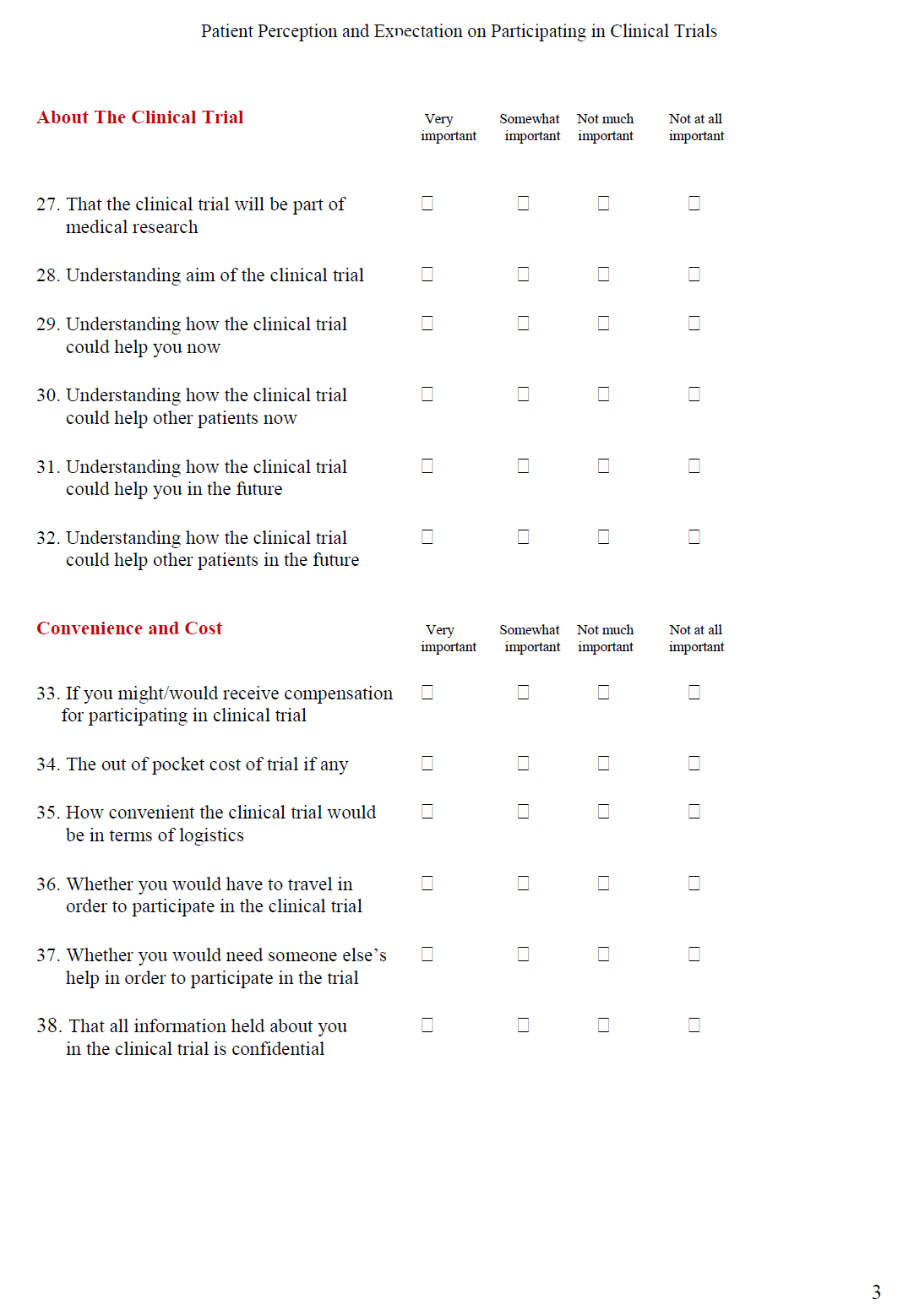


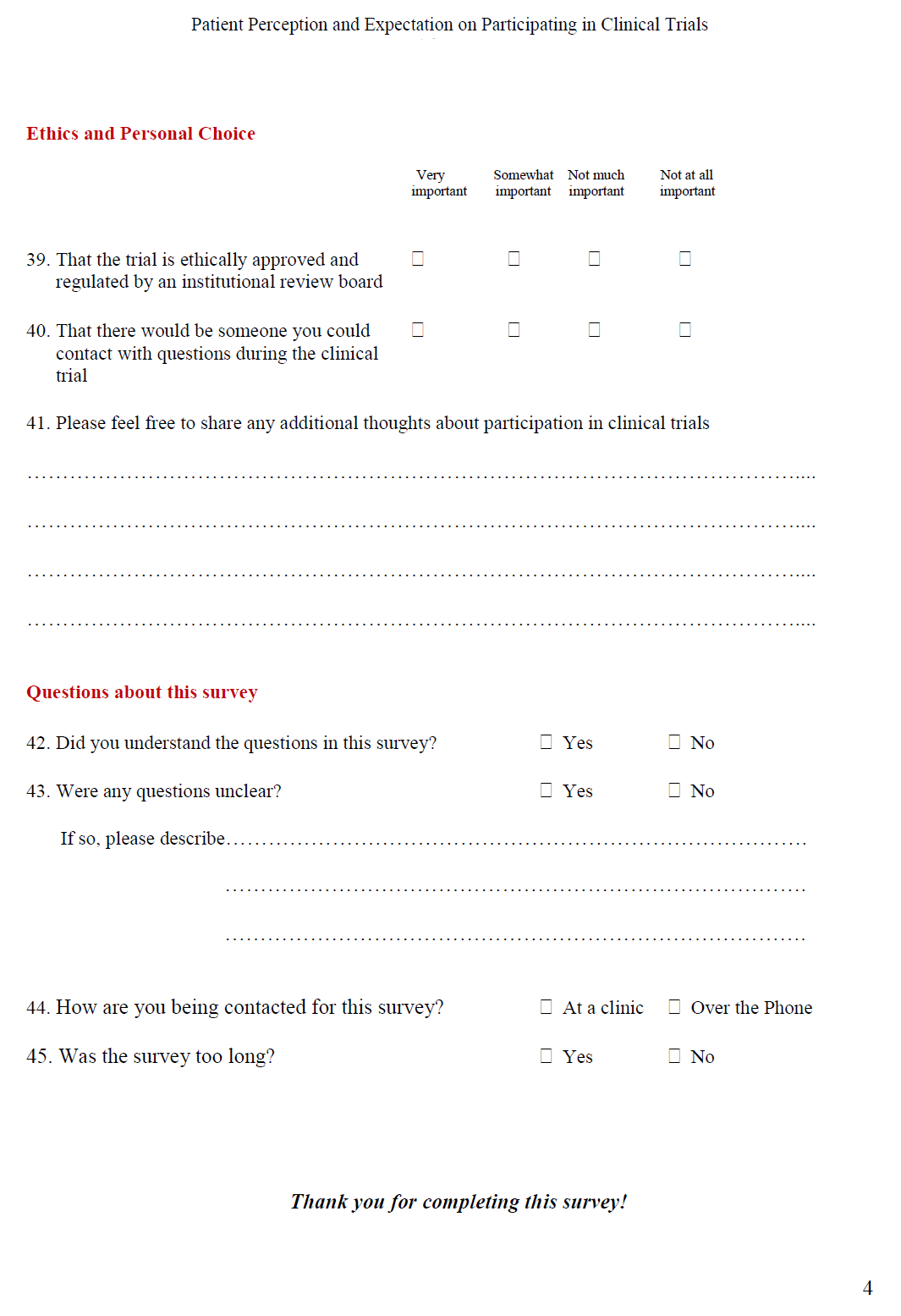

Supplement: Supplementary file 1 — Additional file 1. Patient_questionnaire. [file 13104_2019_4724_MOESM1_ESM.docx]
